# Orthogonal projection to latent structures and first derivative for manipulation of PLSR and SVR chemometric models' prediction: A case study

**DOI:** 10.1371/journal.pone.0222197

**Published:** 2019-09-25

**Authors:** Fatma F. Abdallah, Hany W. Darwish, Ibrahim A. Darwish, Ibrahim A. Naguib

**Affiliations:** 1 Pharmaceutical Analytical Chemistry Department, Faculty of Pharmacy, Beni-Suef University, Alshaheed Shehata Ahmad Hegazy St., Beni-Suef, Egypt; 2 Department of Pharmaceutical Chemistry, College of Pharmacy, King Saud University, Kingdom of Saudi Arabia; 3 Analytical Chemistry Department, Faculty of Pharmacy, Cairo University, Kasr El-Aini St., Cairo, Egypt; 4 Department of Pharmaceutical Chemistry, College of Pharmacy, Taif University, Al-Hawiah, Taif, Saudi Arabia; Newcastle University, UNITED KINGDOM

## Abstract

Novel manipulations of the well-established multivariate calibration models namely; partial least square regression (PLSR) and support vector regression (SVR) are introduced in the presented comparative study. Two preprocessing methods comprising first derivatization and orthogonal projection to latent structures (OPLS) are implemented prior to modeling with PLSR and SVR. Quantitative determination of pyridostigmine bromide (PR) in existence of its two associated substances; impurity a (IMP A) and impurity b (IMP B); was utilized as a case study for achieving comparison. A series consisting of 16 mixtures with numerous percentages of the studied compounds was applied for implementation of a 3 factor 4 level experimental design. Additionally, a series consisting of 9 mixtures was employed in an independent test to verify the predictive power of the suggested models. Significant improvement of predictive abilities of the two studied chemometric models was attained via implementation of OPLS processing method. The root mean square error of prediction RMSEP for the test set mixtures was employed as a key comparison tool. About PLSR model, RMSEP was found 0.5283 without preprocessing method, 1.1750 when first derivative data was used and 0.2890 when OPLS preprocessing method was applied. With regard to SVR model, RMSEP was found 0.2173 without preprocessing method, 0.3516 when first derivative data was used and 0.1819 when OPLS preprocessing method was applied.

## Introduction

**Fig 1:**
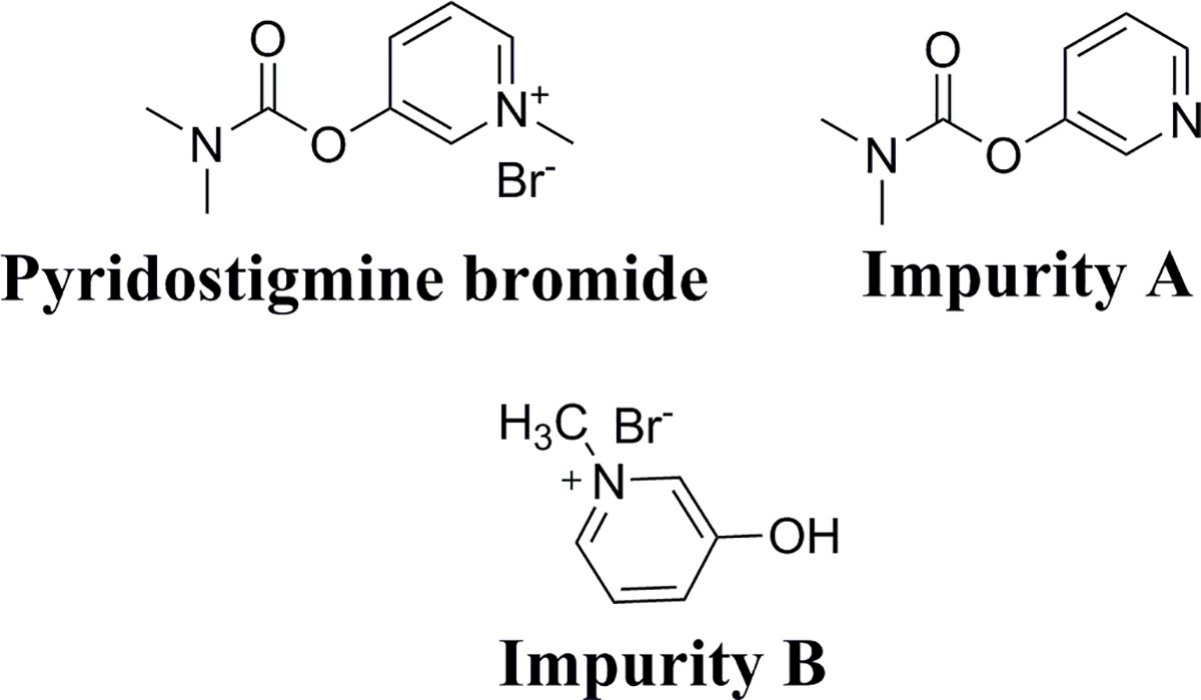
The chemical structure of Pyridostigmine bromide (PR), impurity A (IMP A) and impurity B (IMP B).

Pyridostigmine bromide (PR) is chemically known as 3-[(dimethylcarbamoyl) oxy]-1- methylpyridinium bromide [1,2], **Fig 1**. It is the best medication in case of the myasthenia gravis [3,4] due to its reversible choline esterase inhibition effect and parasympathomimetic effect [5]. The broadest spread of the drug was in the first gulf war as a safeguard of soldiers from chemical weapons like nerve gases [6].

Various methods concerned with analysis of PR were revealed upon deep research in literatures encompassing spectrophotometry [7], HPLC [8–11], GC [12–15] and CE [16–18]. Furthermore, several HPLC methods [19–23], radioisotopic techniques [24–26] and green HPTLC method [27] were illustrated to verify the purity profiling of PR. Moreover, previousely developed HPTLC method was described for stability studies and determination of PR in existence with its associated degradation product [28]. Regarding the literature survey, no reported spectrophotometric methods were established to assess PR and its associated substances. Impurity A; Pyridin-3-yl-dimethylcarbamate (IMP A) and impurity B; 3-Hydroxy-N-methylpyridinium bromide (IMP B) are the related substances of PR with reference to the British pharmacopeia BP [1]. They also proved to be the main inactive metabolites of PR [23]. Additionally, IMP B is also its alkaline-induced degradation product [28].

In the pharmaceutical industry, analysis of degradation products and process-related impurities is an critical function. The prospect of toxic effects or even side effects and reduced effectiveness of active ingredients must be lowered to a minimum level. Subsequently, pharmacopoeias and ICH guidelines promoted establishing of very restrictive requirements for proportions of impurities in pharmaceutical products. The major analytical challenge was the massive variance between the proportions of active ingredients and impurities, thus the analytical method should have an adequate selectivity and be able to simultaneously analyze the target analyte and its impurities [29]. Chemometric methods are potential alternative approach for instantaneous estimation of multicomponent pharmaceutical mixtures due to quick data collection utilizing rapid scanning spectrophotometers. Former illustration od the basic principles and application of PLS and also SVR was found in details [30].

Orthogonal projection to latent structures (OPLS) is a relatively new method for preliminary handling of data. Systematic flactuations of the spectral data are canceled via OPLS; faciliting the translation process of the results. On the other hand, the employment of first derivative data has been recently studied in the implementation of chemometric analysis [31] and the removed variations could be subjected to further analysis to give more knowledge [32].

The developed work was devoted to provide a comparative study for the results of PLSR and SVR models employing original first derivative and OPLS preprocessed data. The presented research involved six chemometric models namely; PLSR, DPLSR (PLSR coupled with first derivative data), OPLS-PLSR (PLSR coupled with OPLS preprocessed data), SVR, DSVR (SVR utilizing first derivative data) and OPLS-SVR (SVR utilizing OPLS preprocessed data).

## Experimental

### Instrument

UV-1601 model UV–visible double beam spectrophotometer (SHIMADZU, Japan) model PC with quartz cell of 1 cm and UV–PC personal software version 3.7 was utilized. The width of spectral band is 1 nm and 2800 nm min^-1^ is the speed of wavelength-scanning.

### Samples

#### Pure samples

Pyridostigmine bromide and IMP A were purchased from Sigma-Aldrich Chemie GmbH, Germany, their purities were investigated to be 99.98% and 99.90 for PR and IMP A, respectively, according to the reference method [1] for PR and the published HPLC method [19] for IMPA. Alkaline degradation of PR under specified condition was done resulting in IMP B [27, 28] with purity of 99.80% according the published HPLC method [19].

#### Pharmaceutical formulation

Each tablet of Mestinon^®^ (batch no. 80085169) is claimed to provide 60 mg of PR by its producing company; Switzerland gmbh, Birsfelden, Switzerland.

### Chemicals and solvents

Methanol with HPLC grade was imported from Sigma-Aldrich Chemie GmbH, Germany.

### Solutions

#### Standard solutions

Stock standard solutions (1 mg mL^-1^) of PR, IMP A and IMP B were made using methanol.Methanol was then used to dilute stock solutions accurately to make their respective working solutions (100 μg mL^-1^). Both stock and working solutions were freshly prepared and kept in refrigerator to be reused within 24 h.

### Procedures

#### Linearity

**Fig 2:**
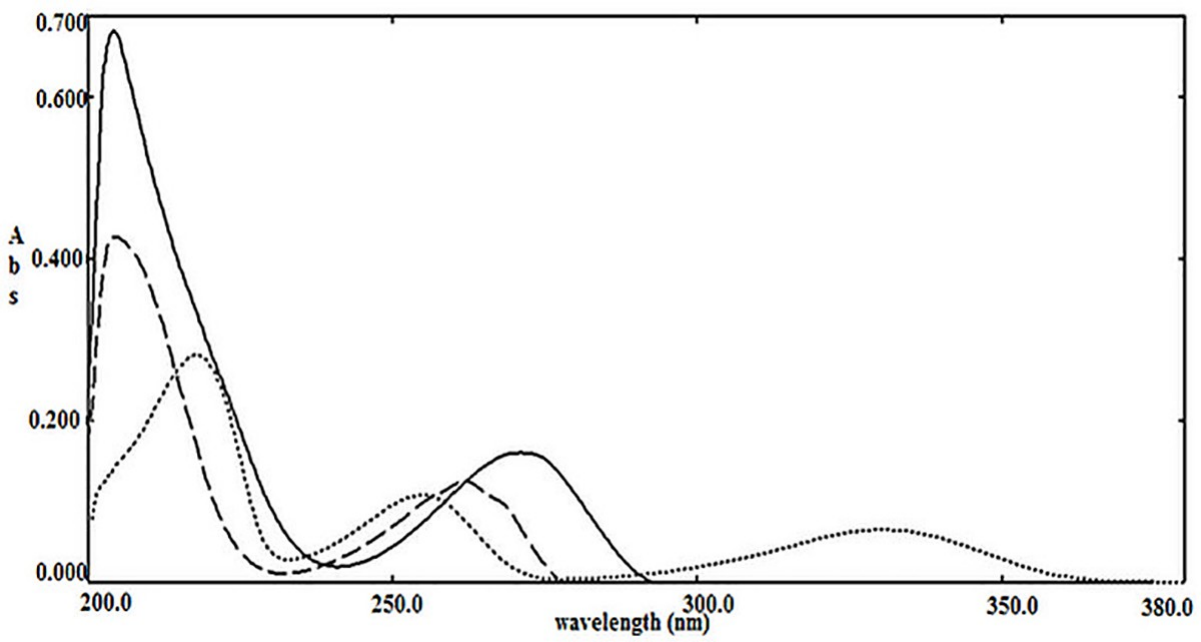
Zero order absorption spectra of 10 μg mL^-1^ of PR (____), 10 μg mL^-1^ of IMP A (-----), 10 μg mL^-1^ of IMP B (……) using methanol as a blank.

UV spectra of the three compounds under study were scanned from 200 to 350 nm. The ranges of PR, IMP A and IMP B were shown to be 5–70 μg mL^-1^, 5–60 μg mL^-1^ and 5–50 μg mL^-1^, respectively. The linearity was revealed at their corresponding λ_max_ (270 nm, 262 nm and 329 nm for PR, IMP A and IMP B, respectively). By application of beer-Lambert’s law basing on the mean of three spectra of different concentrations, extinction coefficients were calculated for all at each nanometer in this range [151 data points]. The scanned spectra of the studied ingredients with concentration of 10 μg mL^-1^ for all are shown in **Fig 2**.

### Experimental design

#### Calibration and test sets

The calibration set composed of the main drug and its associated substances (IMP A and IMP B) were designed as a 4 level 3 factor calibration design employing 4 concentration levels coded as –2, –1, +1 and +2. The central level associated with each compound is represented by the level coded +1. About PR, cocentrations of 20 μg mL^-1^, 30 μg mL^-1^, 50 μg mL^-1^and 60 μg mL^-1^ were codeded by –2, –1, +1 and +2, respectively. Concerning IMP A 0.4 μg mL^-1^, 0.6 μg mL^-1^, 1 μg mL^-1^and 1.2 μg mL^-1^ were coded by–2, –1, +1 and +2, respectively. With regard to IMP B,–2, –1, +1 and +2 codes refered to concentations of 0.5 μg mL^-1^, 0.7 μg mL^-1^, 1.1 μg mL^-1^and 1.3 μg mL^-1^, respectively. The main objective of the design is to confirm ultimate spanning for the mixtures in space; as there are 4 mixtures for each component at every level of concentration producing 16 mixtures to provide the training set μ[13]. The central levels of the design were 50 μg mL^-1^, 1 μg mL^-1^ and 1.1 μg mL^-1^, respectively, for PR, IMP A and IMP B. The concentration of every level for every compound was determined on the basis of its calibration range and also on the fact that concentrations of IMP A and IMP B in the design were involved in about 3% determined with respect to molar basis of the main drug to provide a wide range of possibilities for future analysis. The optimum preprocessing method which provided accurate results for the studied models was investigated to be mean centering of data. The freshly prepared mixtures of the independent test set were employed to prove the the validity and predictive ability of the promoted chemometric models. For development of the independent test set, five mixtures of the training set were selected and freshly prepared in addition to preparation of another four independent mixtures within the concentration space of the design. **Table 1** represents the concentration design matrix for both calibration and test sets.

**Table 1:** The 4 level 3 factor experimental design of 16 training set mixtures together with the 9 test set mixtures shown as concentrations of the mixture components in μg mL^-1^.

Mixture No.	Training set			Test set		
	PR	IMP A	IMP B	PR	IMP A	IMP B
**1**	20	0.4	0.5	20	0.6	0.7
**2**	20	0.6	0.7	50	1	0.7
**3**	30	0.6	1.3	50	0.6	1.1
**4**	30	1.2	0.7	20	0.4	0.5
**5**	60	0.6	0.5	30	1	0.5
**6**	30	0.4	1.1	45	0.5	0.8
**7**	20	1	1.1	25	0.7	0.5
**8**	50	1	0.7	30	0.8	0.5
**9**	50	0.6	1.1	25	0.5	0.6
**10**	30	1	0.5			
**11**	50	0.4	1.3			
**12**	20	1.2	1.3			
**13**	60	1.2	1.1			
**14**	60	1	1.3			
**15**	50	1.2	0.5			
**16**	60	0.4	0.7			

#### Application to pharmaceutical formulation *(*Mestinon^®^ tablets)

Twenty tablets of **Mestinon**^**®**^ were weighed, shattered and then finly-powdered. The well- powdered tablets were mixed homogenously. Then an accurately weighed amount of the prepared powder equal to 100 mg of pure PR was carefully placed inside 100-mL volumetric flask and then 75 mL methanol was poured into the flask. Ultimate solubility of the active pharmaceutical ingredient into methanol was provided via continuous ultrasonication of the prepared flask for half hour. The hot ultrsonicated solution was allowed to cool at the room temperature. Finally, methanol was carefully poured to complete the solution to the mark to give 1000 μg ml^-1^ stock solution. Filtration and dilution of the solution with methanol were done subsequently to provide 100 μg ml^-1^ working solution.

Aliquot equivalent to 1 mL of the working solution was transferred to 10 ml-volumetric flask and the accurate volume was adjusted via dilution with methanol. The average of three respective spectra was stored. Six times repetitions of the experiment were done then the resulted spectra were processed by the proposed suggested models.

#### Software

The codes for the SVR algorithm were downloaded from the internet website http://onlinesvr.altervista.org/. Codes for PLSR (PLS1 algorithm [32]), bootstrap and grid search for optimum SVR parameters were described in details in lab using Matlab^®^ 7.1.0.246 (R14). All calculations were performed using a dual core CPU, 4.00 GHz, 4.00 GB of RAM under Microsoft Windows Seven. OPLS codes were written by H. Li and downloaded from MathWorks website (http://www.mathworks.com/matlabcentral/fileexchange/47767-libpls-1-95-zip/content/libPLS_1.95/opls.m, Jan 2016).

## Chemometric methods

The basic concept of multivariate calibration models is finding a relation between the spectra in the data matrix ***X*** and the concentrations in a data vector ***c***. For constructing a multivariate calibration model, various methods were developed. The most common ones are multiple linear regression (MLR), principal component regression (PCR) and partial least squares regression (PLSR). PCR and PLSR can deal with a large number of spectral variables via decomposing the ***X*** data into a relatively small numbers of what is known as the scores. The scores matrix ***T*** then replaces the original ***X*** matrix in the subsequent regression steps [33, 34].

### Partial least squares regression (PLSR)

Mathematical basis of PLSR results in PLS components number (latent variables LVs) from decomposition of predictor matrix ***X*** and the response vector ***c*** [30, 32] according to the following equations:

X=T.P+E (1)

c=T.q+f (2)

***T*** and ***P*** are, respectively, the scores and loadings for ***X***, ***q*** is the loading vector for ***c***, and ***E*** and ***f*** are the residuals for ***X*** and ***c***, respectively. PLSR is commonly implemented in the industry. Furthermore, several applications reported that PLSR is superior to principle component regression PCR which motivate us to insert this method in this comparative study.

#### Optimization of number of latent variables for the PLSR model

Randomly splitting the training set into two thirds and one third; namely, bootstrap training set and bootstrap test set, respectively, via bootstrap technique which predict how many optimum number of PLS components are [35, 36]. Establishing the PLSR model via the bootstrap training set to predict the bootstrap test set samples and calculating the error of prediction were clarified by this equation

$$RMSEP=\sqrt{\frac{1}{N}\sum_{n=1}^{N}{\left(C_{n}-C_{n}^{A}\right)}^{2}}$$ (3)

Where N is the number of bootstrap test set samples, *C*_*n*_ is the known concentration for sample n and ${\hat{C}}_{n}^{A}$ is the corresponding predicted concentration at a given number of PLS components. **Eq (3)** represents just one iteration. Increasing the number of iterations clearly permits picking up all samples in both training and test set data, consequently 1000 iterations were utilized in this study. For optimum selection of PLS components, the average of the 1000 root mean square error of prediction (RMSEP) values for different number of PLS components was plotted against the corresponding number of components. For bootstrap training set, mean centering was applied every time.

### Support vector regression (SVR)

Consider a data set *X* ***(****I × J****)*** and an output vector *c*. Finding a multivariate regression function f(***x***) based on ***X*** by using a sample spectrum is the objective to predict a required output feature such as a concentration of chemical compound. Equations of SVR are clearly explained in the literature [37, 38] and summarized in the following equation

$$f\left(x\right)=\sum_{ij=1}^{N}(\alpha_{i}-\alpha_{i}{}^{*})\langle Ø\left(x_{i}).Ø(x_{j}\right)\rangle +b$$ (4)

where *α*_*i*_ and *α*_i_^*^ are the Lagrange multipliers satisfying the necessity 0 ≤*α*_*i*_, *α*_i_*≤ *C*. *C* is a supplemental parameter named the penalty error or regularization constant which define the trade-off between the model simplicity and training error. A comprehensive description of Eq (4) and the parameters *a* and *C* are illustrated in the literature [38–40]. The parameter *b* is the substitute of the regression function f(*x*). ε-insensitive loss function is an additional necessary factor widely applied for SVR and will be studied and optimized in our study [41, 42]. The ability to handle linear data and also non-linear ones through kernels is a valuable characteristic of SVR. In the introduced work, linear SVR model was applied, where preplanned experimental design was constructed to guarantee linearity of spectral data. In the prediction step, the validity of the optimum model was examined, where an unknown $\hat{c}$ value can be given as follows [43]:

$$\hat{c}=\sum_{i=1}^{I}(\alpha_{i}-\alpha_{i}{}^{*})x_{i}^{\prime }x_{j}+b$$ (5)

#### Optimization of the linear SVR model parameters

An implementation of a grid search based on 4-fold cross validation provided the optimum values for ε and *C* to give the lowest root mean square error of cross validation (RMSECV). The primary range of values for ε was (0.01–1) and for C (30–1000). With each set of SVR parameters, 4 samples (N = 4) were eliminated, the remaining 12 (I–N) samples were processed by a linear SVR model, predicting the RMSECV for the N samples that was eliminated, and then the average of RMSECV after all samples were removed was computed as follows

$$\mathrm{RMSECV}=\sqrt{\frac{1}{I}\sum_{i=1}^{I}{\left(c_{i}-{\hat{c}}_{i}\right)}^{2}}$$ (6)

Where *c*_*i*_ is the true concentration for sample *n* and ${\hat{c}}_{i}$ is the corresponding predicted concentration.

### Preprocessing methods

#### First derivatization

Recently, the combination of derivative techniques with multivariate calibration methods has been proposed [31]. Bagtash et al [31] mentioned that first derivatization overcomes the spectral overlapping and the best recoveries values were resulted after combination of derivative techniques with PLS model. According to the presented study, the autoprediction results were improved after coupling of PLSR with first derivative technique comparing to PLSR with respect to RMSEC.

#### Orthogonal projection to latent structures (OPLS)

Orthogonal projections to latent structures method (OPLS) is a relatively newly introduced method for data preprocessing. It removes variation from X (descriptor variables; spectral data) that is not correlated to Y (property variables; concentration of PR in our case). In mathematical expressions, it removes systematic variation in X that is orthogonal to Y. Full description of the mathematical explanation and proper application of the method is provided in literature [44, 45].

Both chemometric methods (PLSR and SVR) were applied on zero order absorption spectra, fist derivative spectra and OPLS-spectra to construct a fully informative chemometric comparison.

## Results

### PLSR and SVR parameters

**Fig 3:**
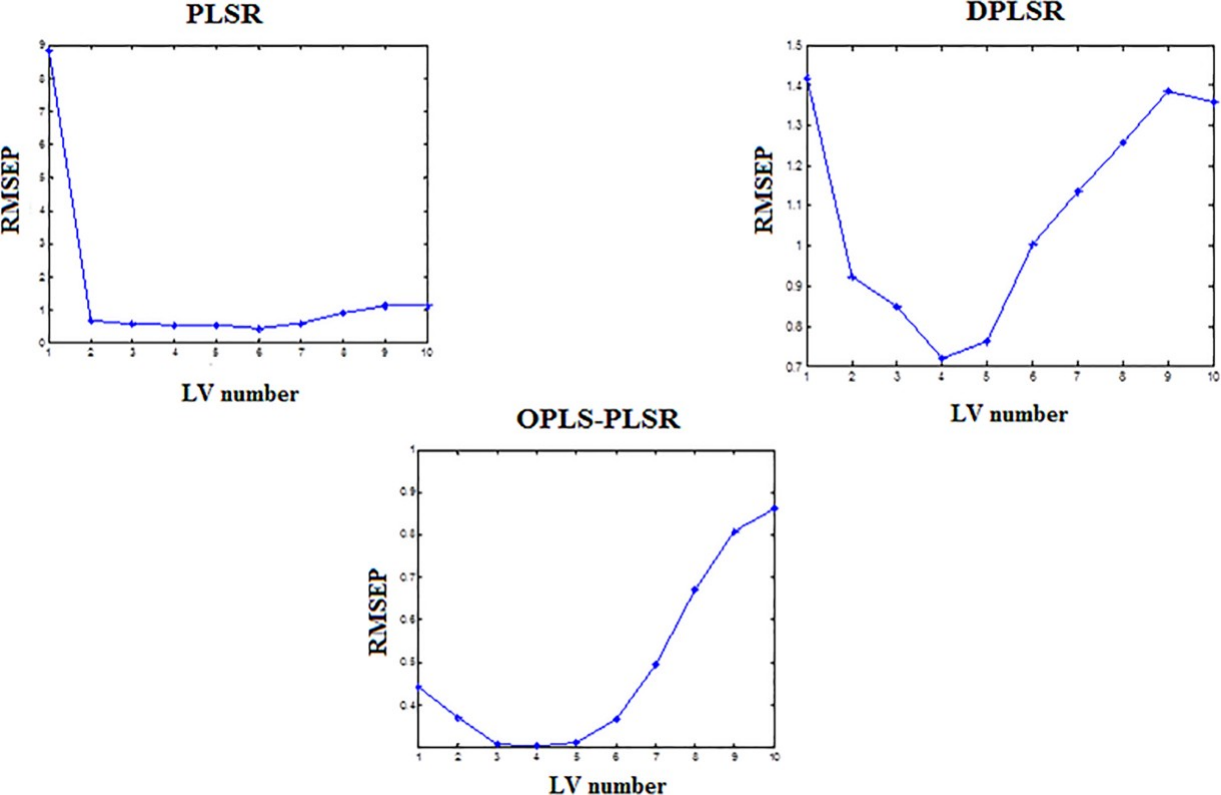
Selection of the optimum number of the latent variables (LVs) via plotting the number of PLS components versus the corresponding root mean square error of prediction (RMSEP) by using the bootstrap technique.

The optimum number of PLS components chosen for establishing the calibration model for the training set to determine PR by bootstrap technique was 2 for PLSR, 4 for DPLSR and 3 for OPLS-PLSR, **Fig 3**.

For optimum SVR parameters, the lowest RMSECV (Eq (6)) which was given by the grid search resulted in (e = 0.15 and C = 220), (e = 0.36 and C = 990) and (e = 0.21and C = 120) for SVR, DSVR and OPLS-SVR methods respectively.

### Data analysis results

**Fig 4:**
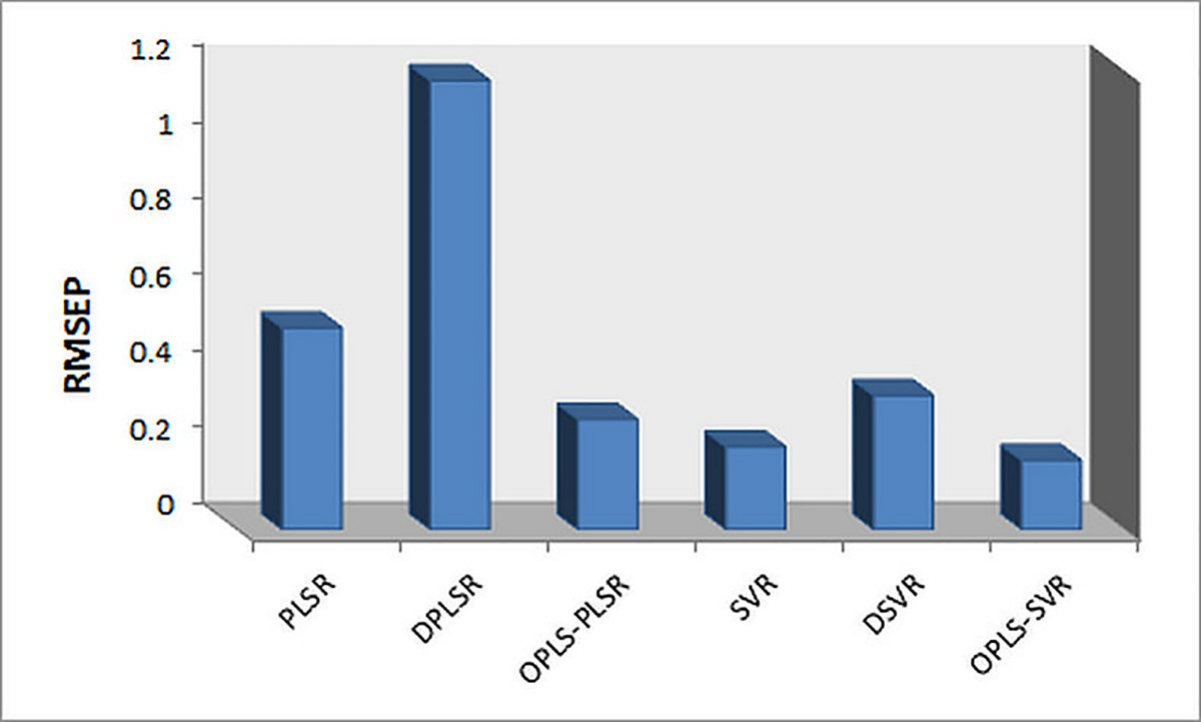
RMSEP plots for the prediction of independent test set samples for PR using the proposed models: 1-PLSR, 2-DPLSR, 3- OPLS-PLSR, 4- SVR, 5- DSVR and 6- OPLS-SVR.

Structural similarity of PR and its related substances cause their high overlap in UV spectra as illustrated in **Fig 2**, exhibiting difficulty in analysis of such mixture by applying univariate approaches. Six methods of multivariate calibration (PLSR, DPLSR, OPLS-PLSR, linear SVR, DSVR and OPLS-SVR) were compared in the presented work. These methods were applied to protend the concentrations of PR in both of training and test sets; the prediction results are given in **Table 2** and **Table 3**, respectively. To assess models’ predictive abilities, the RMSEP was selected as a parameter; RMSEP comparative plot for prediction of test samples is shown in **Fig 4**. It is evident that the developed chemometric methods could be applied for determination of the target analyte in its tablets eliminating any interference from tablets’ excipients. The results were compared with those obtained from the reference method [1] and no significance differences was found in terms of the accuracy and precision (**Table 4)**.

**Table 2:** Analysis results for the prediction of the training set (autoprediction) by the proposed chemometric methods for calibration.

Training set	PLSR		DPLSR		OPLS-PLSR		SVR		DSVR		OPLS-SVR	
Taken (μg mL^-1^)	Found (μg mL^-1^)	%R	Found (μg mL^-1^)	%R	Found (μg mL^-1^)	% R	Found (μg mL^-1^)	% R	Found (μg mL^-1^)	% R	Found (μg mL^-1^)	% R
20	19.45	97.25	19.06	95.28	19.63	98.15	19.85	99.25	19.34	96.70	19.77	98.87
20	19.90	99.52	20.08	100.39	20.02	100.1	20.15	100.75	20.36	101.8	20.13	100.63
30	30.09	100.31	30.56	101.88	30.20	100.67	30.29	100.98	30.64	102.14	30.21	100.70
30	30.53	101.75	29.96	99.87	30.26	100.87	30.15	100.50	30.25	100.82	30.21	100.70
60	59.60	99.33	59.38	98.96	59.89	99.82	59.88	99.81	59.29	98.82	59.79	99.65
30	30.08	100.27	29.99	99.97	30.11	100.37	30.15	100.49	30.17	100.57	30.15	100.51
20	19.83	99.15	20.08	100.41	19.97	99.85	19.85	99.25	19.64	98.2	19.83	99.17
50	50.23	100.45	50.63	101.27	50.18	100.36	50.07	100.14	50.54	101.09	50.04	100.09
50	49.94	99.89	50.46	100.92	50.04	100.08	50.07	100.14	50.36	100.72	49.95	99.89
30	30.57	101.90	30.25	100.84	30.40	101.33	30.15	100.50	30.36	101.2	30.21	100.70
50	49.44	98.88	50.10	100.21	49.56	99.12	49.62	99.25	50.01	100.01	49.51	99.019
20	19.92	99.61	19.97	99.84	19.83	99.15	19.85	99.25	20.12	100.61	19.79	98.97
60	59.87	99.79	60.01	100.02	60.01	100.02	60.09	100.16	59.67	99.44	59.86	99.76
60	59.84	99.73	60.01	100.01	59.94	99.9	60.00	100.00	59.64	99.4	59.79	99.65
50	50.33	100.66	50.03	100.06	49.56	99.12	50.08	100.17	49.64	99.28	50.11	100.23
60	60.37	100.62	59.43	99.05	60.38	100.63	60.15	100.25	59.36	98.93	60.21	100.35
**Mean** **(%)**		**99.94**		**99.94**		**99.97**		**100.06**		**99.98**		**99.93**
**S.D**		**1.070**		**1.399**		**0.767**		**0.540**		**1.373**		**0.639**
**RMSEC**		**0.8350**		**0.4891**		**0.2920**		**0.1671**		**0.4369**		**0.2097**

**Table 3:** Analysis results for the prediction of the independent test set by the proposed chemometric methods for validation.

Test set	PLSR		DPLSR		OPLS-PLSR		SVR		DSVR		OPLS-SVR	
Taken (μg mL^-1^)	Found (μg mL^-1^)	%R	Found (μg mL^-1^)	%R	Found (μg mL^-1^)	% R	Found (μg mL^-1^)	% R	Found (μg mL^-1^)	% R	Found (μg mL^-1^)	% R
20	19.72	98.59	19.39	96.96	19.74	98.71	20.12	100.58	20.28	101.38	20.05	100.27
50	49.96	99.93	50.58	101.15	50.58	101.16	50.24	100.48	50.36	100.72	50.21	100.42
50	49.19	98.37	50.24	100.49	49.78	99.57	49.61	99.22	49.64	99.28	49.79	99.58
20	19.60	97.98	19.06	95.28	19.59	97.96	19.82	99.10	20.36	101.80	19.79	98.95
30	30.63	102.10	30.48	101.61	30.31	101.05	30.10	100.34	29.64	98.80	30.21	100.7
45	44.29	98.42	45.18	100.41	44.95	99.89	45.09	100.21	44.64	99.20	44.79	99.53
25	24.53	98.11	27.47	109.88	24.93	99.74	24.89	99.58	25.36	101.44	24.87	99.48
30	29.31	97.70	32.09	106.95	29.92	99.74	29.92	99.73	30.36	101.20	29.89	99.62
25	24.97	99.89	25.23	100.94	24.83	99.34	24.64	98.54	25.36	101.44	24.79	99.16
**Mean** **(%)**		**99.01**		**101.52**		**99.68**		**99.75**		**100.58**		**99.75**
**S.D**		**1.320**		**4.242**		**0.949**		**0.664**		**1.094**		**0.556**
**RMSEP**		**0.5283**		**1.1750**		**0.289**		**0.2173**		**0.3516**		**0.1819**
**LVs**		**2**		**4**		**3**						

**Table 4:** Statistical comparison of the results obtained by the proposed methods and the reference method for the determination Pyridostigmine bromide in Mestinon tablets^®^.

Parameters	PLSR	DPLSR	OPLS-PLSR	SVR	DSVR	OPLS-SVR	Reference method**
**Mean**	98.71	99.20	99.24	99.79	98.76	99.49	99.97
**SD**	0.913	0.932	1.273	1.542	0.995	1.414	0.941
**Variance**	0.834	0.870	1.620	2.377	0.825	2.000	0.866
**n**	6	6	6	6	6	6	6
Student’s t-test*(2.228)	0.029	0.111	0.164	0.414	0.033	0.270	_________
F- test*(5.050)	1.062	1.019	1.828	2.684	1.073	2.258	_________
**One-way ANOVA test** **F-critical (4.9503)** **P-value**	**0.880** **0.506**						

* Figures in parenthesis are the corresponding tabulated values at *p* = 0.05.

** Direct spectrophotometric determination at 269 nm [1].

## Discussion

Coupling of the traditional chemometric methods; PLSR and SVR with OPLS and first derivatization as preprocessing methods is recently introduced and studied in our work.

The present study describe a fully- informative comparison between six chemometric models (PLSR, DPLSR, OPLS-PLSR, SVR, DSVR and OPLS-SVR) via their use in analysis of different mixtures of PR and its related substances (IMP A and IMP B). The high similarity in the chemical structures of the investigated compounds was behind the high overlap in their UV spectra (**Fig 2)**. This overlap makes their simultaneous analysis by the traditional univariate approaches of handling of UV data is very difficult. Accordingly, multivariate approach was more potential alternative for their simultaneous analysis.

Concerning results of auto prediction of PLSR-based models (PLSR, DPLSR and OPLS-PLSR); coupling of PLSR with fist derivatization (DPLSR) provided auto prediction results which are better than that of PLSR with original data, but the best results were obtained after coupling of PLSR with OPLS with respect to root mean square error of calibration RMSEC. On the other hand, RMSEC of SVR model utilizing original data is the lowest comparing to the other five models, so no influence was detected on the auto prediction results after coupling of SVR with first derivatization (DSVR) or OPLS (OPLS-SVR) with respect to RMSEP. The values of RMSEC of DSVR and OPLS-SVR were still acceptable.

The predictive ability of the chemometric model is presented by the root mean square error of prediction (RMSEP) of the test set. Concerning PLSR-based methods, coupling of PLSR with first derivatization improved the recoveries values of the test set, but the best results were obtained after coupling of PLSR with OPLS with respect to RMSEP. With regard to SVR-based models (SVR, DSVR and OPLS-SVR), RMSEP of OPLS-SVR is the lowest comparing to the other five models, but no significant effect was detected on the prediction results after coupling of SVR with first derivatization (DSVR) with respect to RMSEP.

Comparing the prediction results of test set for the six proposed methods with each other, OPLS-SVR has the lowest RMSEP then SVR reflecting highest ability of SVR-based method to handle future samples and then OPLS-PLSR method, **Table 2**.

A set of conclusive remarks could be observed and highlighted from the above mentioned discussion. According to many published researches, PLSR is the most applicable model in chemometrics and has several applications in pharmaceutical industry overcoming PCR and multivariate linear regression MLR [30]. It was revealed that the SVR possessing higher predictive power than PLSR in many case studies [30]. Coupling of the traditional PLSR chemometric model with OPLS as a preprocessing tool provide higher predictive ability than PLSR, so it can be applied instead of the complicated SVR model keeping the advantage of simplicity of PLSR model and providing high predictive ability comparative to SVR model.

Finally, the six established methods were successfully implemented for assessment of PR in Mestinon^®^ tablets. These methods offered additional advantages over the existing HPLC methods [19–23] such as cost effective and time-saving. The results of analysis of Mestinon^®^ obtained by studied methods were compared to the reference one [1] statistically. The tabulated t and F values were more than the automatically calculated ones proving that the significant difference was generally absent regarding both of accuracy and precision. One way ANOVA test was applied for statistical analysis of the results obtained by the proposed methods and the reference method. The test ascertains that the proposed methods are comparable and as precise and accurate as the reference method, **Table 4.**

It is evident that the proposed methods could be used for quantitative determination of PR in its bulk material and pharmaceutical tablets; keeping the advantages of spectrophotometric methods for quantitative determination of samples with minimum sample preparations, economic laboratory consumption and cheap materials.

## Conclusion

The present study compared six different models for multivariate calibration methods and highlighting novel manipulations of these methods. The six models were PLSR, DPLSR, OPLS-PLSR, SVR, DSVR and OPLS-SVR that were compared using a pharmaceutical UV dataset as a case study. For prediction ability of the future samples, values of RMSEP of independent test set reveal that OPLS-SVR was the best one followed by SVR and OPLS-PLSR. For comparing results and routine analysis, the 4 level 3 factor design has been confirmed as an efficient and economical. The results revealed that these models were selective and accurate procedures in quality control analysis of PR without hindrance from its related substances. Furthermore, the novel manipulations of the traditional chemometric methods can be employed for further pharmaceutical research studies using simple and cost-saving instruments like UV spectrophotometer even if the number of interfering components is high and spectra of them are severely overlapped.
